# Nursing students’ experiences of mentorship by NGRNs on placement: a qualitative descriptive study

**DOI:** 10.1177/17449871261466811

**Published:** 2026-08-14

**Authors:** Sheetal Sood, Kath Peters, Lauretta Luck

**Affiliations:** Associate Lecturer, School of Nursing and Midwifery, Western Sydney University, Penrith, NSW, Australia; Professor, School of Nursing and Midwifery, Western Sydney University, Penrith, NSW, Australia; Associate Professor, Director, Centre for Nursing and Midwifery Research, Western Sydney University’s School of Nursing and Midwifery/Nepean Blue Mountains Local Health District, Penrith, NSW, Australia

**Keywords:** clinical placement, mentors, mentorship, new graduate registered nurses, nursing, nursing education, qualitative research, students

## Abstract

**Background:**

The shortage of experienced nurses within the global workforce has resulted in healthcare systems increasingly relying on inexperienced nurses to meet workforce demands. These demands include the mentorship of undergraduate nursing students by newly graduated registered nurses. However, there is little understanding of how new graduate nurses filling mentorship roles impacts on students’ clinical placement experience.

**Aim:**

The study explored pre-registration nursing students’ experiences of mentorship by new graduate registered nurses (NGRNs).

**Method:**

The study used a qualitative descriptive design underpinned by naturalistic inquiry. Data were collected using semi-structured interviews with pre-registration nursing students who were mentored by NGRNs. Twelve participants were recruited via social media using purposive and snowball sampling until data sufficiency was achieved. Braun and Clarke’s Thematic Analysis framework guided data analysis.

**Results:**

Interviews generated three themes and five sub-themes: Theme one – More than placement: A sense of belonging and included two sub-themes (i) Breaking the ice: Being welcoming and approachable and (ii) Cultivating empathy and emotional connection: *She never judged me*. Theme two – Pedagogical approaches of NGRNs in teaching with sub-themes (i) Collaborative learning in the clinical setting: *We are learning together*; (ii) The role of time and patience: *Do not rush, take your time* and (iii) Building confidence and promoting independence: *She allowed me to be hands on*. Theme three – Barriers to effective student learning.

**Conclusion:**

NGRNs formed a positive relationship in the clinical setting by being welcoming, approachable and inclusive with students. This study prompts a reconsideration of established assumptions that experienced mentors provide the most effective mentorship. Relational attributes enhance students’ learning and confidence, highlighting the need to re-evaluate current mentorship models and recognise the positive impact these mentors may have on the future nursing workforce.

## Introduction

Nurses represent 59% of the health workforce globally, forming the largest occupational group within healthcare services (World Health Organisation [WHO], 2020). The nursing profession faces an unprecedented workforce challenge, with WHO (2020) projecting a global shortfall of 4.5 million nurses by 2030. To meet the projected shortage by 2030, the number of nurse graduates would need to increase by an average of 8% annually, while simultaneously retaining these graduates. In addition, demographic trends intensify this shortage, with an estimated 17% of the global nursing workforce expected to retire within 10 years; therefore, 4.7 million new nurses will need to be educated and employed to maintain current nursing workforce numbers (Buchan, 2022).

Due to projected nursing workforce gaps, and to meet future healthcare demands, it is essential to increase nursing student numbers and reduce the high attrition rates among nursing students (Diane et al., 2023; Doleman et al., 2022; Patterson et al., 2017). There are many factors contributing to nursing students leaving the profession, such as changes in career plans, family or health commitments (Diane et al., 2023; Jack et al., 2018). However, one critical aspect to address is a negative clinical placement experience which may also be linked to students withdrawing from the course (Canzan et al., 2022; Jack et al., 2018; Jones-Berry, 2018; ten Hoeve et al., 2017).

While completing clinical placement, students are supervised by registered nurses who work with them on a day-to-day basis. These nurses are allocated a variety of titles which are often used interchangeably and include mentor, preceptor, facilitator, clinical supervisor or buddy nurse (Brown et al., 2020; Ford et al., 2016; Rebeiro et al., 2017; Ryan et al., 2022). Regardless of terminology, registered nurses who work with pre-registration nursing students play a significant role in fostering a successful learning experience (Bowen et al., 2019). However, some students struggle to develop effective relationships with their mentors which can negatively affect their learning experience (Brown et al., 2020). Since these relationships directly influence both clinical placement experiences and learning outcomes, their contribution to positive clinical experience cannot be underestimated (Cusack et al., 2020). Given global nursing workforce demands, it is important to create and foster a supportive clinical learning environment facilitated by effective mentorship.

For this study, the term ‘mentorship’ or ‘mentor’ refers to registered nurses who supervise nursing students during clinical placements. The term ‘nursing students’ refers specifically to undergraduate pre-registration nursing students. This study is focused on pre-registration nursing students mentored by new graduate registered nurses (NGRNs). An NGRN, also referred to as a novice registered nurse, is a registered nurse in their first 12 months of full-time (or equivalent) clinical practice (Nijkamp et al., 2024).

### NGRNs as mentors

Existing knowledge about the mentoring experience has developed from several studies involving experienced nurses supporting students in the clinical environment (Lee and Chiang, 2021; Pedregosa et al., 2020; Tuomikoski et al., 2018). Typically, experienced nurses provide mentorship; however, workforce shortages and the limited availability of experienced mentors have led to increasing reliance on NGRNs to support students’ learning. Anyango et al. (2024) conducted a study exploring the career choices of final-year nursing students, in which students worked with NGRNs due to the limited availability of experienced mentors within the clinical environment. While the study highlighted students’ mixed feelings about the quality of supervision, it offered limited insights into their actual experiences. Similarly, in the Australian context, McKenna et al. (2018) used a qualitative descriptive approach to examine the teaching activities undertaken by new graduate nurses. The findings provided valuable insights into the experience of NGRNs in a teaching role, but it did not specifically discuss the students’ experience (McKenna et al., 2018). Despite their lack of clinical experience, it is important to recognise NGRNs offer recent knowledge of the student nurse experience. This transition from experienced-led mentorship to near-peer mentorship raises important questions about how mentorship by NGRNs is enacted in practical settings. Therefore, qualitative research is needed to explore how NGRNs’ mentorship influences student nurses’ clinical learning experiences.

## Aim

The aim of the study was to explore pre-registration nursing students’ experiences of mentorship by NGRNs. Specifically, it explored the benefits and challenges for students when being mentored by NGRNs.

## Methods

### Theoretical framework

This study was underpinned by Lincoln and Guba’s (1985) naturalistic inquiry (NI) that enables the researcher to focus on describing individuals, situations and experiences. NI is particularly suitable when there is limited information about the phenomenon under study. NI explores subjective and complex experiences as they unfold naturally, allowing in-depth analysis of emerging themes from participants’ subjective experiences (Lincoln and Guba, 1985).

### Research design

A qualitative descriptive design consistent with NI was utilised to describe mentorship as experienced by pre-registration nursing students and gain a better understanding of the phenomenon.

### Participants/recruitment

Participants were pre-registration nursing students enrolled in a pre-registration Bachelor of Nursing programme in an Australian university who had been mentored by an NGRN and were a minimum of 18 years of age.

Participants were recruited by posting a recruitment flyer on social media sites (Facebook™ and Instagram™) between February and May 2025. In addition, information was posted on the Australian College of Nursing website and through a virtual student portal used by a nursing school at an Australian university. Participants were recruited using purposive and snowball sampling. Pre-registration nursing students who were interested in participating in the study contacted the researcher directly via email.

### Data collection

The first author (S.S.), a female university academic and registered nurse, conducted all interviews for the project supported by two senior nurse academics with doctoral qualifications. Data were collected through individual semi-structured zoom interviews, using open-ended questions to facilitate discussion. All interviews were audio-recorded and transcribed verbatim. The interview guide was informed by the literature and reviewed and discussed with expert qualitative researchers (supervisors). Each interview lasted between 15 and 30 minutes. Data collection continued until data sufficiency was reached, which occurred after twelve interviews. According to LaDonna et al. (2021) sufficiency depends on both the rigour of the analytical process and the richness of the data. The sample size in this study was guided by the principles of data sufficiency and information power. Rather than aiming for numerical saturation, the focus was on collecting information-rich data focusing on the research topic, adequately reflecting the experiences of nursing students, thereby contributing to new insights (Malterud et al., 2016). This approach ensured that the information gathered was comprehensive enough to answer the research question.

### Ethical considerations

The Western Sydney University Human Research Ethics Committee (HREC) (HREC Approval Number H16423) approved this study. The study ensured the protection of participants’ rights, privacy and confidentiality throughout the research process. All participants provided voluntary informed verbal consent to participate in the study, ensuring autonomy. No participants withdrew from the study. To ensure anonymity and confidentiality, pseudonyms were assigned to all participants. Although none of the participants experienced any distress during the interviews, a list of contact details of free professional counselling services was provided to participants via the participant information sheet.

### Data analysis

Data analysis was conducted concurrently with data collection using reflexive thematic analysis as outlined by Braun and Clarke (2022). The first author read and re-read the interviews and repeatedly listened to the audio-recordings. Initial thoughts and codes were then manually generated using a Microsoft Word™ document with coloured highlights. The first author then worked through each dataset, and individual data from the transcripts were then matched to the generated codes. The data were coded and developed into potential themes. Coding was iterative and interpretive rather than based on coder agreement. The three authors met regularly to discuss the development of themes. Discussions among authors were used to deepen reflexive engagement with the data and refine conceptual themes. Once consensus was reached, three main themes were finalised to accurately reflect their content and meaning (see Table 1 for an example of the analytical process).

**Table 1.** Exemplar of analytical process.

Themes	Sub-themes	Categories	Codes
More than placement: A sense of belonging	Breaking the ice: Being welcoming and approachable	Creating welcoming environment	*‘With NG I feel like I was welcomed. You do not feel like you are walking behind them and bothering them throughout the day. I felt that I was heard and seen’.* (Shyla)
		Approachable	*‘Every one of the new grads that I approached were very friendly, down to earth and so willing to help’.* (Victoria)
	Cultivating empathy and emotional connection: *She never judged me*	Culture of empathy	*‘When I approach the NG, I found they were more empathetic and supportive especially with the student.* (Victoria)
		Emotional connection	*‘they would introduce themselves and they will talk about when they were at the university themselves’*. (Victoria)
		Feeling safe	*‘She never judged me that I don’t know anything’.* (Bindu)
Pedagogical approaches of NGRNs in teaching	Collaborative learning in the clinical setting: *We are learning together*	Inclusive learning environment	*‘They include you in the learning process, okay we can go learn this new thing together. Whenever the educator teaches the NG, it is very beneficial for me as well’.* (Shyla)
		Reflective learning	*‘I want you to read this and go through it, and then we will talk about it later. She would ask me a lot of questions. And at the end of our shifts, we will do like a reflection which really helped with my professional growth’.* (Natalie)
	The role of time and patience: *Do not rush, take your time*	Time and patience	*‘NG was patient and gave me time. Keep on saying do not rush take your time. I would rather go to the NG because they are more patient with me’.* (Shyla)
		Willingness	*‘All of them are so happy and willing to teach you all of their knowledge that they have learned. She actually stood by my side every step of the way’.* (Natasha)
	Building confidence and promoting independence: *She allowed me to be hands on*	Presence	*‘She was there when I did the first time. She checked it three or four times and after that she gave me the space to do it on my own’.* (Bindu)
		Reassurance	*‘they give you that reassurance that it is ok to make a mistake, and this is how you grow’.* (Nathan)
		Space to grow	*‘She also gave me, like, a lot of independence, like she knew that if I was capable of doing something she wasn’t hovering over me and like you know, pick at every little thing, which I really hated’.* (Brenda)
Barriers to effective student learning		Limited clinical experience	*‘NGs are technically students. They are still learning. I don’t think students should be paired with NGRN’.* (Sandy)
		Feeling unsupported	*‘It was kind of hard to be confident in what I was doing as she wasn’t confident in herself as well and it was her first month of the NG program, so it is understandable’.* (Natalie)
		NGRN–staff relationship	*‘My learning with NGRN I would say very little compared to senior nurses. I don’t know how supported NGs themselves in some settings are. I wouldn’t say much, no’.* (Sandy)
			*‘She (NGRN) was kind of intimidated by senior nurses. A NGRN who was not confident in herself, just puts students in uneasy and uncomfortable position’.* (Natalie)

NGRN: new graduate registered nurse.

### Rigour and reflexivity

To demonstrate rigour, principles of credibility, transferability, dependability and confirmability were applied (Lincoln and Guba, 1985). Credibility was supported by reflexivity. The first author is a registered nurse with prior experience mentoring students. A reflexive diary was maintained to document assumptions, beliefs and reflections that could potentially influence the research process. Managing pre-conceived assumptions was considered paramount as the study was informed by the researcher’s own experiences and background (Okoko et al., 2023). In addition, regular peer debriefing with supervisory team members helped monitor the researcher’s thoughts and beliefs, across each phase of the research process. Confirmability was further enhanced through the inclusion of direct participant quotes in the findings (Cope, 2014). Transferability was supported by providing detailed descriptions of sampling, the recruitment process, data collection and analysis methods. Dependability was established through the maintenance of a detailed log of the research process and decisions made at each stage regarding collection and analysis. This study used the Consolidated Criteria for Reporting Qualitative Research (COREQ) guidelines (Tong et al., 2007).

## Findings

Twelve pre-registration nursing students participated in the study. Ten participants were female and two were male. Eight were third year, two were second year, and two students were in their first year of the pre-registration nursing programme. The participants’ ages ranged from 19 to 40 years (mean age 29.5 years). Five participants were employed on a casual basis in the healthcare sector, and one had nursing experience in another country. Students undertook clinical placement in a variety of settings (see Table 2). The participant sample reflects considerable diversity in terms of age, year of study and prior nursing experience. Although the sample is predominately females, it includes both sexes and covers multiple clinical areas within acute care settings.

**Table 2.** Participants’ demographic data.

Participant pseudonym	Age	Gender	Year in nursing	Previous nursing experience	Clinical placement area
Laila	22	Female	Third year	Casual AIN	Paediatric ward/Surgical ward
Shyla	30	Female	Second year	Overseas RN	Colorectal ward
Victoria	40	Female	Third year	Aged care support worker	Day surgery ward
Bree	20	Female	Third year	Casual AIN/NDIS worker	Rehab ward
Bindu	19	Female	First year	N/A	Day surgery/Rehab ward
Nathan	20	Male	Third year	N/A	Surgical ward
Shelly	30	Female	First year	N/A	Medical ward
Sandy	19	Female	Third year	Casual AIN	Recovery area
Natalie	26	Female	Second year	N/A	Surgical ward
Natasha	21	Female	Third Year	N/A	Surgical ward
Brenda	20	Female	Third Year	N/A	Neurology/Oncology
Nelson	24	Male	Third year	Casual AIN	Gastro/Cardiology

AIN: assistant in nursing; RN: registered nurse; NDIS: national disability insurance scheme.

Participants’ experiences are presented within themes and sub-themes (see Table 3). These themes illustrate students’ experiences working with NGRNs, who provided a safe environment that encouraged learning and growth through time and patience. However, there were also challenges associated with NGRNs mentoring students.

**Table 3.** Themes and sub-themes.

1. More than placement: A sense of belonging	1.1 Breaking the ice: being welcoming and approachable. Cultivating empathy and emotional connection: *She never judged me*
2. Pedagogical approaches of NGRNs in teaching	2.1 Collaborative learning in the clinical setting: *We are learning together* 2.2 The role of time and patience: *Do not rush, take your time* Building confidence and promoting independence: *She allowed me to be hands on*
3. Barriers to effective student learning	

NGRN: new graduate registered nurse.

### Theme 1. More than placement: A sense of belonging

Participants highlighted that one benefit of being mentored by NGRNs was that they created a safe space. Examples from participants illustrated that NGRNs who were friendly, welcoming, approachable, empathetic and supportive enhanced their sense of belongingness in the clinical environment. Feeling a sense of belonging affected how students connected and engaged with their mentors. This theme has two sub-themes: (i) Breaking the ice: Being welcoming and approachable and (ii) Cultivating empathy and emotional connection: *She never judged me* elucidating the two main attributes of mentors that affected participants’ clinical placement experience.

#### Breaking the ice: Being welcoming and approachable

This theme revealed the importance of mentoring relationship between students and the NGRN. ‘Breaking the ice’ represents the NGRN’s concerted effort to foster a supportive environment and make students comfortable. All participants commented on the welcoming and approachable nature of NGRNs. They valued the feeling of being welcomed. Shyla (2nd year) commented: ‘*With (new graduate) I feel like I was welcomed. You do not feel like you are walking behind them and bothering them throughout the day. I felt that I was heard and seen*’. Participants reported feeling grateful when mentors made the effort to check-in and see how students were managing. Victoria stated (3rd year): ‘*Every one of the new grads that I approached were very friendly, down to earth and so willing to help. They all time asking us are you ok? Do you need any help*’? This attitude was best summed up with a comment by Shelly (1st year), who was mentored by an NGRN on her first rotation: ‘*I am like, I was praying from then on, every placement that I hope I’ll have an NGRN as my mentor*’.

In this study, feeling unwelcome and unwanted by more senior nurses was a concern raised by some participants. Although NGRNs were described as supportive and inclusive, some senior nurses positioned students as observers rather than active participants. Natasha (3rd year) was frank in her response: *‘They want nothing to do with the students, because students hold them back. They have their own set routine and having a student nurse come along with them. They’re very much like you can just stand back and watch’*.

#### Cultivating empathy and emotional connection: *She never judged me*

The participants in this study highlighted that NGRNs demonstrated empathy towards them, which helped foster a meaningful and supportive mentoring relationship. Participants felt comfortable asking questions, making mistakes and they felt listened to without feeling that they were being judged by their mentors. Participants reported that NGRNs were able to relate to the students’ experiences by drawing their own recent transitions into practice, which helped them understand the students and the challenges they faced.

Throughout the interviews participants highlighted the importance of positive relationships with their mentors. Most importantly sharing academic knowledge and their own individual experiences was highly appreciated by the students. Victoria (3rd year) explained that: ‘*They would introduce themselves and they will talk about when they were at the university themselves*’.

Participants felt a sense of safety with the NGRN, due to the empathetic connection established in this mentoring relationship, as indicated by Victoria (3rd year): ‘*When I approached the NG, I found they were more empathetic and supportive, especially with the student. I feel like it is very important to be on the same emotional level*’. Participants also discussed their expectations of mentorship and voiced concerns about feeling judged by their mentors. However, when working with an NGRN Bindu comment: ‘*She never judged me that I do not know anything*’ resulting in Bindu (1st year) feeling safe to ask questions. Bindu, further emphasised that mentors who were supportive and made them feel wanted positively influenced their experience.

### Theme 2: Pedagogical approaches of NGRNs in teaching

The participants attested to mentors using a diversity of teaching strategies to support students’ learning styles to promote positive learning experiences. They used innovative approaches such as collaborative learning, creating learning opportunities, thereby enhancing students’ confidence. Having recently transitioned from student to NGRN, shared experiences such as studying and undertaking clinical placements facilitated the connection between NGRNs and the students. Within this theme, three sub-themes were identified: (i) Collaborative learning in the clinical setting: *We are learning together*; (ii) The role of time and patience: *Do not rush, take your time*; and (iii) Building confidence and promoting independence: *She allowed me to be hands on.*

#### Collaborative learning in the clinical setting: *We are learning together*

Participants appreciated the interpersonal relationship they had with the NGRN which enhanced their collaborative learning. They worked collaboratively with the NGRN, learning from each other and developing their knowledge and skills. An advantage of NGRNs as mentors was that both groups benefitted from this mentoring relationship as they searched for information together and they both learnt. As mentioned by Shyla (2nd year): ‘*It is like very beneficial to have an NG and a student together. It is great for both sides learning; we are both learning together at the same time*’.

Throughout the interviews, there were many examples where participants agreed that learning was a mutual process because NGRNs were simultaneously learning and developing as nurses while mentoring students. Shyla (2nd year) further supports this: *‘They include you in the learning process, okay we can go learn this new thing together. Whenever the educator teaches the NG, it is very beneficial for me as well’.*

Another highly valued learning and teaching strategy used by NGRNs was their proactive mentoring, particularly asking reflective questions to deepen students’ understanding. Natalie (2nd year) further emphasised this experience, stating: *‘I want you to read this and go through it, and then we will talk about it later. She would ask me a lot of questions. And at the end of our shifts, we will do like a reflection which really helped with my professional growth’*.

#### The role of time and patience: *Do not rush, take your time*

Participants consistently highlighted the importance of time and patience in their mentoring relationship with NGRNs. Being mentored by an NGRN fostered a sense of confidence in them as they advanced through their clinical placement, mainly because NGRNs were willing to slow down, offer guidance and create a relaxed learning environment. Participants felt more comfortable and at ease when NGRNs were patient and took the time to explain procedures thoroughly, which enhanced their learning: *‘NG was patient and gave me time. Keep on saying do not rush take your time. I would rather go to the NG because they are more patient with me’*. (Shyla 2nd year)

The willingness of NGRNs to extend help and give them time made participants feel more comfortable, as mentioned by Shyla (2nd year): ‘*Do not rush, finish with this one and I will help with the other one*’. This was well supported by the following statement by Natasha (3rd year): ‘*All of them are so happy and willing to teach you all of their knowledge that they have learned. She stood by my side every step of the way*’.

In addition to being patient and providing time, accessibility was another important factor to the participants which made them feel comfortable thereby supporting their learning process: *‘I think I learned a lot of things when I was with her, and I probably wouldn’t have learned if I was with a senior nurse because of just how busy they are. It also was more I think she was around more for me than some of the other RNs that I was buddied up’*. (Brenda 3rd year)

Participants commented that NGRNs were able to break down concepts to explain procedures such as administering medications to the students. Shelly (1st year) conveyed: ‘*She walked me through each step by step, and that was like a new thing. She took her time to go through medication with me*’, after which Shelly reported having a better understanding of the proper sequencing of checks of medication administration.

#### Building confidence and promoting independence: *She allowed me to be hands on*

Beyond creating a comfortable learning environment, NGRNs promoted independence, critical thinking and confidence among participants when performing clinical skills. Students were offered space and a chance to practice skills with appropriate supervision, which helped build their confidence and critical thinking. Brenda (3rd year) appreciated the independence given to her: ‘*She also gave me, like, a lot of independence, like she knew that if I was capable of doing something she wasn’t hovering over me and like you know, picking at every little thing, which I really hated*’.

Bindu, a first-year nursing student also talked about how she was given a chance to experience and to learn a skill, and once confident, the NGRN provided her the space where she was able to perform the skill on her own, describing a gradual increase in responsibility: ‘*She was there when I did the first time. She checked it three or four times and after that she gave me the space to do it on my own*’.

Providing participants a sense of reassurance was very much appreciated by Nathan (3rd year) as mentioned in his statement: ‘*They give you that reassurance that it is ok to make a mistake, and this is how you grow*’.

### Theme 3: Barriers to effective student learning

While participants in this study identified the benefits of working with NGRNs, they also experienced challenges such as time constraints, limited experience and workload pressures imposed upon this mentoring role. Staff shortages meant senior nurses were not always available to mentor them, and allocation to NGRN was often not a purposive choice but a necessity. Experienced nurses were viewed as ‘gold standard’, despite all the positives highlighted above. As mentioned by Shyla (2nd year): ‘*If there is not an experienced RN on the floor incharge sometimes allocate me to the NGRN*’.

NGRN’s lack of experience and confidence to teach were also affected by environmental factors. Certain clinical areas, such as critical care, may not be an ideal setting for NGRNs to mentor students due to the complexity and demands of the clinical environment. Given that NGRNs are still learning and developing their own clinical competence, students in these placements may not receive the level of support and guidance they need. Laila (3rd year) shared her experience of being treated differently when buddied with an NGRN in a critical care area. She explained how the NGRN did not really have time for her, so she felt like a burden to them. She noted that it was difficult to ask questions: ‘*I always felt afraid to ask for help because I know how much stress the NG has to intake. She also felt like a lot of pressure on herself*’. Sandy (3rd year) working with NGRN in high acuity setting further emphasised this point by highlighting that: ‘*NGs are technically students. They are still learning. I don’t think students should be paired with NGRN*’.

Individual factors such as NGRNs being in their first rotation or lacking confidence in their own practice were identified as potential barriers to effective student learning. Participants spoke of not feeling confident when working with NGRNs on their first rotation. The first rotation can be overwhelming, making it hard to take on an additional responsibility of mentoring, as stated by Natalie (2nd year): ‘*It was kind of hard to be confident in what I was doing as she wasn’t confident in herself as well, and it was her first month of the NG program, so it is understandable*’. Conversely, Sandy (3rd year) talked about her experience of working with NGRN in her second rotation. She stated: ‘*NG was towards the end of her rotation. So, it didn’t really feel like she was a NG as she was very confident*’. Shyla (2nd year) also talked about how she was disadvantaged by working with an NGRN in her first rotation: *‘Whenever I am budded with the NG all I can do is the vitals observation, just helping them with the wash, shower, making the bed and all. They advise us not to administer medication with the NGRNs’*.

As discussed in theme two reflective learning techniques was highly valued as it helped to consolidate student centred learning during clinical placement. However, the same strategy was not appreciated by Nelson (3rd year) highlighting preferences in learning styles as he conveyed: *‘I know he was trying to help, but it’s like torture, asking me questions. Like I am a student and have my own way of learning, but I think he was trying to prove himself which does not help with my learning’.*

Additionally, it was perceived that some individual nurses did not feel confident or ready to take on a mentoring role. Limited knowledge among some NGRNs was identified as one of the challenging aspects that hindered participants’ clinical learning. Although they appreciated the benefits of learning alongside NGRNs, participants also felt they were still developing their clinical skills and would have preferred to work with more experienced nurses. Laila (3rd year) stated: ‘*I also want to work with senior nurses, they have more knowledge compared to NGRNs*’. According to Nathan (3rd year): ‘*NGs are adapting, and they are learning too. They are very limited in the kind of educational aspect. They do not have exact educational experience as other RNs*’. Some participants perceived NGRNs to be insecure when supervising nursing students, as mentioned by Bree (3rd year): *‘I feel they get nervous knowing that they have a student with them, but I believe they have knowledge and skills. So, give them a chance to help students. I think maybe it will grow their confidence’*.

Despite these challenges, having NGRNs involved in this mentoring role was favourably appraised by most of the participants. Participants highlighted the benefits to be mentored by both NGRNs and experienced nurses suggesting a team-based approach to mentorship. However, participants felt systemic barriers such as NGRNs not being adequately supported in the role. Participants perceived a lack of support from senior staff made it difficult for NGRNs to provide effective mentorship. Their lack of confidence can indirectly impact on the learning experience for students. As stated by Natalie (2nd year): ‘*She (NGRN) was kind of intimidated by senior nurses. An NGRN who was not confident in herself, just puts students in uneasy and uncomfortable position*’. Reflecting on her learning experience Sandy (3rd year) also shared a similar experience stating: ‘*My learning with NGRN I would say very little compared to senior nurses. I don’t know how supported NGs themselves in some settings are. I wouldn’t say much, no*’.

## Discussion

This discussion is built on the findings that highlight NGRNs’ empathy, approachability and willingness to spend time with students, contributing positively to their sense of belonging and learning experiences. Non-judgemental attitude of NGRNs helped students feel safe, understood and emotionally supported. However, although participants acknowledged NGRNs were excellent mentors for students, they expressed concerns regarding their limited clinical experience and knowledge, particularly in high-acuity settings. This discussion critically explores these findings, focusing on the interconnected themes of belongingness, inclusive learning for student success and participants’ perspectives on mentorship challenges.

### Feelings of belongingness

The need to feel a sense of belonging on clinical placement is common among nursing students (Squire et al., 2024). Human relationships, particularly those between nursing students and their mentors, are essential components of successful clinical placements (Rodríguez García et al., 2014). The quality of this relationship plays a significant role in determining the success of the learning experience. Seminal research suggests that forming meaningful connections satisfies the powerful and fundamental human need to belong (Baumeister and Leary, 1995). This sense of belonging drives motivation and influences how individuals think, feel and behave within their personal context, both emotionally and cognitively (Baumeister and Leary, 1995; Honda et al., 2016). When students feel safe and experience a sense of belonging, their learning in the clinical environment is enhanced. In such contexts, the desire to feel accepted outweighs the importance of achieving high levels of clinical competency during placement (Borrott et al., 2016; Levett-Jones and Lathlean, 2009).

Participants felt comfortable asking NGRNs questions without fear of judgement. They conveyed that it was easier to seek support from NGRNs, rather than senior staff. As emphasised by Niederriter et al. (2017), qualities such as availability, approachability and willingness to help foster a feeling of comfort with the mentor and thus enhance the learning experience.

### Inclusive learning for student success

NGRNs were simultaneously learning their own clinical skills while mentoring students. They acknowledged when they lacked answers, demonstrating, self-reflection and a collaborative approach to actively seek support from senior nurses, thereby benefitting students’ learning. This resonates with findings from Nielsen et al. (2017) that emphasised being together, doing together and getting along together enhanced students’ learning outcomes.

Collaborative learning can be understood using Vygotsky’s (1978) social constructivist theory, which emphasised the role of social interaction and collaboration in facilitating the development of knowledge. Vygotsky (1978) defined the zone of proximal development as: ‘the distance between the actual development level as determined by independent problem solving and the level of potential development as determined through problem-solving under adult guidance, or in collaboration with more capable peers’ (p. 86). Mentoring between nursing students and NGRNs aligns with Vygotsky’s zone of proximal development. By engaging students through questioning and shared problem-solving, NGRNs facilitated learning within the students’ zone of proximal development, the space where students can achieve greater understanding through a collaborative approach.

However, effective learning also depends on appropriate scaffolding, where the mentor provides support to guide students’ learning process (Chen et al., 2021). The teaching strategy used by the NGRN resonates with the concept of ‘Fading’ in the cognitive apprenticeship model (Collins, 2005: 51), whereby support is gradually withdrawn as the student develop a sense of autonomy and confidence to perform the clinical skill. The participants’ experience also corresponds with findings of Zwedberg et al. (2020), who identified effective mentors as those who provide students with learning opportunities and support them to achieve confidence and independence in performing clinical skills. Although this study by Zwedberg focused on midwifery students, the implications are relevant to nursing students, highlighting the value of this type of support in clinical education. However, it is important to differentiate that, although the students perceived this approach as beneficial, this withdrawal of support did not necessarily reflect an intended teaching strategy. Addressing this distinction is analytically important as intentional fading represents a purposeful teaching strategy, whereas unintentional withdrawal of support carries implications for supervision and learning quality. At the same time, teaching between NGRN and students can be understood as mutually beneficial, where supporting students facilitate learning for NGRNs, thereby reinforcing clinical knowledge and building confidence.

### Students’ perspectives of mentorship challenges

Despite these benefits of collaborative learning as highlighted in theme two, the main study result was participants’ sharing of challenges when the NGRNs lacked the confidence and clinical knowledge to effectively guide students. These challenges may be influenced by the traditional didactic model of learning, where greater value is placed on ‘experienced’ nurse who can directly teach and provide answers. However, this preference may overlook the pedagogical value of collaborative learning approaches, which promote critical thinking, clinical decision-making and active engagement in nursing students (Billett, 2016). This suggests the need to critically analyse the effectiveness of collaborative learning within the context of NGRNs’ developing competence.

Early in their first rotation and working in critical care environments, NGRNs may lack the confidence and clinical competence to effectively mentor students. This aligns with Hampton et al. (2021) and Nijkamp et al. (2024), who noted that NGRNs often experience feelings of frustration and being overwhelmed during their transition into practice. However, after completing the first 6 months of the graduate programme, NGRNs reported feeling more confident and comfortable in their clinical roles (Voldbjerg et al., 2016). This reflects perceptions of participants in the current study where NGRNs in their second rotation were reported to be more confident in mentoring. At this stage, collaborative learning is more effective, as NGRNs gain experience, and becoming more comfortable in the workplace can positively influence the quality of mentorship they provide to the nursing students.

Given the factors that can potentially compromise students’ educational experience, collaborative learning between NGRNs and students should be viewed as context-dependent and careful considerations given to when NGRNs are positioned in these mentoring roles. To navigate this duality, educators and managers can implement a structured mentorship programme, where NGRNs are partnered with experienced RNs who provide guidance, respond to questions and create a safe space that promotes professional growth for both NGRNs and students. This arrangement can provide NGRNs with a trusted mentor who can provide support with clinical practice, patient care and adapting to workplace dynamics (Molle and Allegra, 2021).

## Strengths and limitations

An important strength of this study is that it offers insights into nursing students’ experiences of being mentored by NGRNs, an area that has not been previously explored. The qualitative design provides detailed experiential perspectives. Additionally, the inclusion of a diverse range of participants offers broad representation. Several limitations of this study should be acknowledged and considered when interpreting the findings. Firstly, the participants were self-selected through online recruitment, which may have introduced sampling bias, as students with particularly strong or positive experiences may have been more inclined to participate. Interviews were relatively brief, potentially limiting narrative depth. However, despite this limitation, the study met the criteria for data sufficiency, as rich narratives provided a meaningful understanding of the student’s experience. Data were collected within one national context and predominantly acute care settings, which may reduce transferability to other systems or community settings. As experiences were retrospective, recall bias is possible.

## Implications

These findings highlight mentoring as a key relational component of clinical education, particularly as NGRNs increasingly support nursing students during clinical placements. Mentoring is more effective when NGRNs are supported by experienced nurses, highlighting the value of shared mentoring approaches in clinical practice. Given that NGRNs are increasingly taking on student mentoring responsibilities while managing the same clinical workload as senior nurses, it is essential to support them in this dual role, thereby contributing to the positive learning experience for nursing students. These findings have implications for education and practice, highlighting the need to support NGRNs in mentorship role through targeted training, and implementation of policies that promote team-based mentoring models involving the student, NGRN and experienced nurse fostering shared learning for all. From a policy perspective, NGRNs should not be expected to supervise students until they have completed their preceptorship programme as this allows them to transition more confidently into practice. Further research is needed to build on these findings through larger scale studies, including quantitative research and by exploring the perspectives of both NGRNs and experienced nurses to better understand their experiences and support needs in mentoring roles.

## Conclusion

NGRNs formed a positive relationship with student nurses by being welcoming, approachable, patient, inclusive and creating a non-judgemental environment. These qualities created a safe and supportive environment strengthening emotional connections thereby enhancing students’ learning experience. This study recommends embedding relational mentoring within structured team-based models that support students learning through collaboration with NGRNs and experienced nurses, alongside training and policy directives to ensure NGRNs are supervised and fully supported for mentoring roles. This approach has the potential to enhance learning experience and better prepare the future nursing workforce.

**Key points for policy, practice and/or research**

- NGRNs should complete their preceptorship programme before taking on mentoring roles, enabling more confident transition into practice. NGRNs need professional development opportunities to undertake this mentoring role.
- Facilitating mentoring with NGRNs who are willing and enthusiastic about teaching is better for students’ clinical learning experience.
- Future research could consider NGRNs’ experience of this mentoring role, particularly the type of support they need to develop their skills to provide positive clinical learning experience for students.
- Further research could explore nursing students’ experiences of NGRN mentorship across diverse clinical settings such as aged care, community health and mental health placements.
